# Oxidative Stress and Apoptotic Changes in Broiler Chicken Splenocytes Exposed to T-2 Toxin

**DOI:** 10.1155/2019/5493870

**Published:** 2019-11-25

**Authors:** Yuqi Chen, Shunshun Han, Yan Wang, Diyan Li, Xiaoling Zhao, Qing Zhu, Huadong Yin

**Affiliations:** Farm Animal Genetic Resources Exploration and Innovation Key Laboratory of Sichuan Province, Sichuan Agricultural University, Chengdu, Sichuan 611130, China

## Abstract

T-2 toxin is a trichothecene mycotoxin produced by fungi which are known to contaminate cereals, especially in wheat and corn. T-2 toxin is known to cause a range of toxic effects in humans and animals, including immunosuppression and carcinogenesis. Although the effects of T-2 toxin on condition of chickens' spleens have been reported, there has been no systematic study of damage to the spleen of broiler chickens exposed to T-2 toxin. The purpose of the present study was to assess the effects of T-2 toxin on pathology, rates of apoptosis, oxidative stress, and T-lymphocyte subsets in the spleen of broiler chickens. One hundred and twenty male broiler chickens were randomly assigned to one of four groups (30 birds per group), fed 0 mg/kg (control), 0.5 mg/kg, 1 mg/kg, or 2 mg/kg T-2 toxin, respectively. After 21 days, chickens exposed to T-2 toxin demonstrated decreased relative weight and size of the spleen, increased percentage of apoptotic splenocytes, and evident lesions. Concentrations of reactive oxygen species and MDA content increased in splenocytes during T-2 toxin treatments, whereas activities of SOD, CAT, and GSH-PX decreased. The ratio of CD4+/CD8+ T cells also decreased as the dose of T-2 toxin increased. Overall, these results suggest that T-2 toxin causes oxidative stress, leading to increased rates of splenocyte apoptosis and might impair the splenic immune function of broiler chickens.

## 1. Introduction

Presence of molds in feeds or foods, such as corn or wheat, can result in the production of mycotoxins. Contamination of feed or food by mycotoxins can lead to a diverse array of responses including immunosuppression or carcinogenesis in humans and animals [1]. The T-2 toxin is a type A trichothecene mycotoxin that predominantly contaminates feed ingredients during storage [2]. Prolonged exposure to type A trichothecenes can lead to loss of appetite and weight loss and injury of the oral cavity and esophagus [3]. In addition, the T-2 toxin has been demonstrated to have a radiomimetic effect, inhibiting synthesis of DNA, RNA, or proteins in eukaryotic cells [4]. A number of studies have observed that exposure to T-2 toxin can result in induction of lymphocytic apoptosis in the thymus of mice and Peyer's patches [5], human cervical cancer cells [6], and chicken hepatocytes [7].

It has been suggested that the T-2 toxin is a primary causative pathogen of fatal intestinal tract toxicity in animals and humans, by damaging mucosae and destroying the immune system [8]. Exposure to T-2 toxin has been observed to result in the loss of leukopenia in lymphoid organs and inhibition of erythropoiesis in the spleen and bone marrow [9, 10]. In experimental animals, T-2 toxin causes selective depletion of leukocytes and blood cells, significantly impairs antibody production, and impairs development of dendritic cells [11–13]. Mice orally exposed to 10 mg/kg of T-2 toxin for up to 24 h exhibited increased susceptibility to T-2 toxin of their CD4+ and CD8+ T cells of the thymus [5]. Vlata et al. found that both CD4+ and CD8+ T cells in human peripheral blood lymphocytes treated with T-2 toxin exhibited significant responses after 8 h of exposure, followed by a dramatic decline after 96 h at doses of sublethal (0.1 ng/mL) and lethal doses (10 ng/mL) [14].

Although the effect of T-2 toxin exposure on spleens of chickens has been reported, limited work has been done with the broiler chicken. Therefore, the purpose of the present study was to investigate the effects of exposure of broiler chickens to T-2 toxin on the pathology of the spleen, splenocyte apoptosis, and T-lymphocyte subsets.

## 2. Materials and Methods

### 2.1. Ethical Approval

All experiments were approved by the Animal Ethics Committee of Sichuan Agricultural University (No. 2018-212). Relevant guidelines and regulations were followed for all assays and experiments.

### 2.2. Exposure of Chickens

One hundred twenty 1-day-old ROSS 308 male broiler chickens were purchased from Sichuan Yuguan Agricultural Co., Ltd (Suining, Sichuan, China). After weighing, birds were randomly allocated into 4 groups, each consisting of 6 replicates with 5 birds. Treatment groups received no T-2 toxin (control) (Sigma, Rolla, Missouri, US), 0.5 mg/kg, 1 mg/kg, or 2 mg/kg, respectively. Each experimental replicate was reared in an independent cage at the Teaching and Research Poultry Farm of Sichuan Agricultural University (Ya'an, Sichuan, China). All birds were fed the same feed which met nutritional requirement of ROSS 308 (Table 1). Feed and water were provided *ad libitum* throughout the experiment.

The T-2 toxin was purchased from Sichuan Yuanou Biotechnology Co., Ltd (Chengdu, Sichuan, China). A stock of T-2 toxin was prepared by dissolving in 95% ethanol, mixing with 1 kg basal diet, and then drying. The stock T-2 toxin was diluted to treatment concentrations using the chicken feed to desired concentrations. The T-2 toxin concentrations in feed were confirmed by the use of a ELISA kit (Jiancheng Biotech, Nanjing, Jiangsu, China) as described previously [15].

### 2.3. Sample Collection and Preparation

Following 21 days of T-2 toxin exposure, 6 birds (1 bird per replicate) in each group were randomly selected, and body mass was measured and then euthanized. A total of 6 spleens were collected, and connective tissue was dissected and then weighed. Next, fresh spleen tissues were collected to assess their pathology, rate of splenic apoptosis, and splenic T-cell subsets. A subsample of each spleen was dissected, minced with medical grade scissors, and stored at −80°C for RNA and protein extraction.

### 2.4. Relative Size of Spleen

Ratio of spleen to body weight was calculated for each of the 4 treatments and recorded as R0 (0 mg/kg), R1 (0.5 mg/kg), R2 (1 mg/kg), or R3 (2 mg/kg), respectively. The group R0 was used as the control and used to calculate relative changes in weight of spleens for the R1, R2, or R3 groups, respectively.

### 2.5. Observations of Spleen Pathology

Fresh spleen tissues were fixed with 4% paraformaldehyde overnight and embedded in paraffin. Approximately 5 *μ*m sections were taken from each paraffin-embedded tissue and mounted on a slide, stained by use of hematoxylin and eosin (H&E), and examined using an optical microscope (Olympus, Tokyo, Japan).

### 2.6. Measurement of Apoptosis

A 300-mesh nylon gauze was used to filter the splenocyte suspension. After filtration, splenocyte suspensions were washed using prechilled phosphate-buffered saline (PBS) twice. Cells were resuspended in 1 × binding buffer (BD Pharmingen, Santiago, CA, USA) at a final concentration of 1 × 10^6^ cells/mL. Next, 100 *μ*L of solution was transferred to a culture tube, and 5 *μ*L of PI (BD Pharmingen) and Annexin V-FITC were added (BD Pharmingen), respectively. After mixing, cells were incubated at 25°C for 15 min in the dark, and then 400 *μ*L of 1 × binding buffer (BD Pharmingen) was added to each tube and detected by the use of a flow cytometer (BD Bioscience).

### 2.7. Detection of Splenic T-Cell Subsets

Chicken splenic T-cell subsets were detected as previously described [16]. Following filtration using a 300-mesh nylon gauze, cell suspension was washed twice using prechilled PBS, centrifuged for 5 min at 200 g, and the resultant supernatant was discarded. Pellets were resuspended in PBS, and 100 *μ*L of suspension was transferred to a new culture tube. Cells were incubated with 10 *μ*L mouse antichicken CD4-FITC and CD8-PE (SouthernBiotech, Birmingham, AL, USA), respectively. Following incubation at 25°C for 30 min, 2 mL PBS was added and centrifuged for 5 min at 200 g, and supernatant was discarded. Cells were resuspended in 400 *μ*L of 1 × binding buffer (BD Pharmingen) and measured by the use of a flow cytometer (BD Bioscience).

### 2.8. Real-Time Quantitative Polymerase Chain Reaction (RT-qPCR)

Total RNA was extracted from samples of broiler chicken spleens using Trizol reagent (Takara, Dalian, China). First-strand complementary cDNA was synthesized according to the manufacturer's protocol and stored at −20°C for subsequent real-time quantitative polymerase chain reaction (RT-qPCR). All RT-qPCR reactions were performed in triplicate, and the amplification of extracts was performed as follows: temperature was raised to 95°C for 5 min; next, 36 cycles consisted of 95°C for 10 s, 60°C for 30 s, 72°C for 20 s, then 65°C for 5 s, and finally 95°C for 5 s (BIO-RAD CFX ConnectTM real-time system, Biorad, Hercules, CA, USA). The endogenous gene *β*-actin was used as a reference gene and primers were designed using Primer Premier 5.0: caspase-3 forward: 5′-CATCTGCATCCGTGCCTGA-3′, caspase-3 reverse: 5′-CTCTCGG CTGTGGTGGTGAA-3′; caspase-9 forward: 5′-GCTTGTCCATCCCAGTCCAA-3′; caspase-9 reverse: 5′-CAGTCTGTGGTCGCTCTTGT-3′; *β*-actin forward:5′-CCGCTCTATGAAGGCTACGC-3′, *β*-actin reverse: 5′-CTCTCGGCTGTGGTGGTGAA-3′.

### 2.9. Western Blot

Spleen samples were washed twice using prechilled PBS and centrifuged for 5 min at 3000 rpm and 4°C. Supernatant was discarded and pellets were dissolved in RIPA lysis buffer (Sigma, Rolla, Missouri, USA) containing 1 mM phenylmethanesulfonyl fluoride (PMSF; Sigma) on ice. Next, the lysate was centrifuged at 12,000 rpm for 10 min at 4°C and supernatant was collected. Cellular protein was extracted by the use of the Cell Total Protein Extraction kit (Sangon Biotech, Shanghai, China). The Pierce bicinchoninic acid protein assay kit (BestBio, Shanghai, China) was used to assess the quality of extracted protein. Western blot was performed as previously described by Han et al. [17] using rabbit anti-chicken Caspase-3, Caspase-9, *β*-actin polyclonal (1 : 1500; Abcam, Cambridge, MA, USA), and horseradish peroxidase- (HRP-) labeled IgG secondary antibodies (1 : 800; Abcam). Bands were detected by the use of the enhanced chemiluminescence (ECL) kit (Beyotime, Jiangsu, China) using a CanoScan LiDE 100 scanner (Canon, Tokyo, Japan), and Western blots were analyzed using ImageJ software (v1.80, NIH, USA).

### 2.10. Measurement of Intracellular Reactive Oxygen Species (ROS)

Concentrations of intracellular reactive oxygen species (ROS) were measured using nonionic H2DCFDA, which can cross cell membranes and are then enzymatically hydrolyzed by intracellular esterase to nonfluorescent H2DCF. Following the addition of H2DCFDA, samples were vortexed and incubated at 37°C in the dark for 30 min. Next, samples were washed with PBS and analyzed using flow cytometry (BD Bioscience).

### 2.11. Measurement of Antioxidant Enzymatic Activity and Concentrations of MDA

The activities of superoxide dismutase (SOD), catalase (CAT), and glutathione peroxidase (GSH-PX) and malondialdehyde (MDA) content were measured by the use of kits (Jiancheng Biotech) according to the manufacturer's protocols. All samples were measured in triplicate for each assay.

### 2.12. Statistical Analysis

Statistical analyses were completed by the use of SPSS version 19.0 (SPSS Inc., Chicago, IL, USA). Date are presented as mean ± standard deviation (SD). Differences between control and groups receiving treatment were assessed using one-way analysis of variance and *t*-test, and differences were considered significant at *P<*0.05.

## 3. Results

### 3.1. Relative Size of Spleens

**Figure 1:**
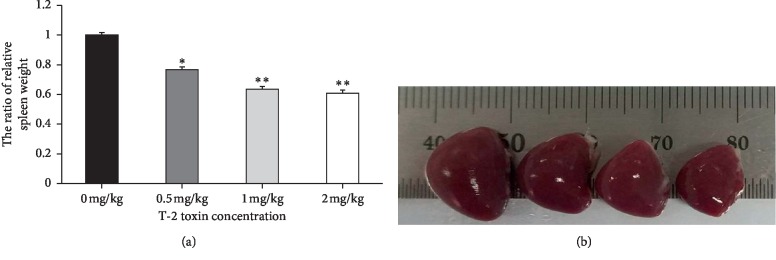
Effects of T-2 toxin exposure on morphology of chickens' spleen. (a) The ratio of relative spleen weight and (b) spleen size. Data are expressed as mean ± sd (*n* = 6). ^*∗*^*P<*0.05, ^*∗∗*^*P<*0.01, as compared to control.

To assess the effect of T-2 toxin treatment on spleen of broiler chickens, relative spleen weight (Figure 1(a)) and size (Figure 1(b)) were compared to control chickens following 21 days of exposure. Overall, an inverse relationship was observed between spleen size of broiler chickens and increasing dosage of T-2 toxin. Relative weight of the spleen in control exposed broiler chickens was significantly greater when compared with all treatments receiving T-2 toxin (*P<*0.05).

### 3.2. Pathology of Spleen

**Figure 2:**
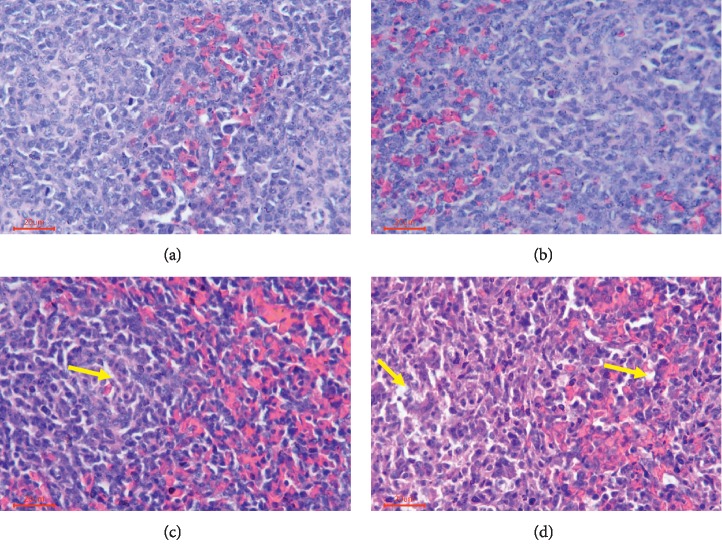
Photomicrographs of H&E stained histological sections of chickens' spleens exposed to T-2 toxin. (a) Structure of control spleen. (b) Congestion of red pulp of spleen in group receiving 0.5 mg/kg T-2 toxin. (c, d) Nuclear debris and vacuoles (yellow arrows) in spleens of broiler chickens receiving 1 mg/kg and 2 mg/kg T-2 toxin, respectively.

Pathological analysis of spleens of broiler chickens exposed to T-2 toxin demonstrated no obvious histopathological lesions following the exposure to 0.5 mg/kg when compared to control exposed broiler chickens (Figures 2(a) and 2(b)). However, broiler chickens exposed to 1 or 2 mg/kg T-2 toxin had increased prevalence of vacuoles and necrotic cells in the splenic corpuscle and splenic periarterial lymphatic sheath when compared to control exposed broiler chickens (Figures 2(c) and 2(d)).

### 3.3. Presence of Apoptotic Cells

**Figure 3:**
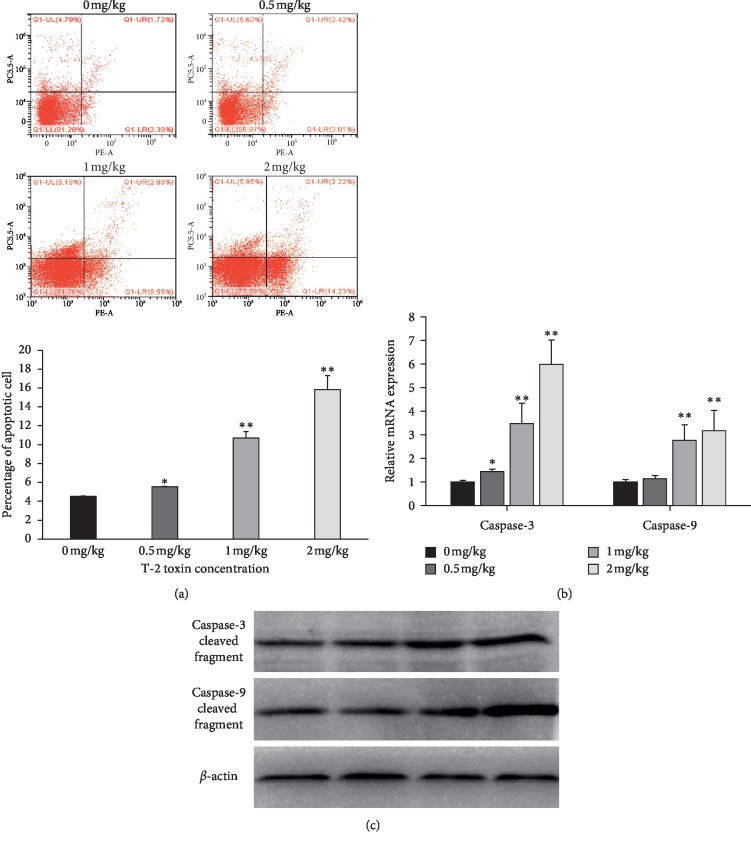
Effect of T-2 toxin exposure on apoptosis of chickens' splenocytes. (a) A scatterplot of apoptotic splenocytes analyzed using flow cytometry following Annexin V and PI staining. (b) Abundances of mRNA and (c) the protein caspase-3 and caspase-9 in splenocytes. Data are expressed as mean ± sd (*n* = 6 independent cell cultures). ^*∗*^*P<*0.05, ^*∗∗*^*P<*0.01, as compared to control.

Field-emission microscopy (FEM) was used to detect apoptotic splenocytes. Rate of splenocyte apoptosis significantly increased with increasing the exposure of broiler chickens to T-2 toxin when compared to control exposed broiler chickens (Figure 3(a)). In addition, abundances of mRNA and proteins of caspase-3 and caspase-9 were monitored to investigate whether apoptosis was induced (Figures 3(b) and 2(c)). Expression of caspase-3 and caspase-9 mRNA was significantly greater in broiler chickens exposed to 1 and 2 mg/kg of T-2 toxin when compared to control (*P<*0.01). Furthermore, broiler chickens exposed to 0.5 mg/kg T-2 toxin had significantly greater abundance of caspase-3 mRNA when compared to control exposed chickens (*P<*0.05). Western blot assessment demonstrated that caspase-3 and caspase-9 protein abundances increased with increasing the dose of T-2 toxin in broiler chickens. Collectively, these results suggest that the exposure of broiler chickens to T-2 toxin results in greater rates of apoptosis in splenocytes.

### 3.4. Concentrations of Reactive Oxygen Species (ROS)

**Figure 4:**
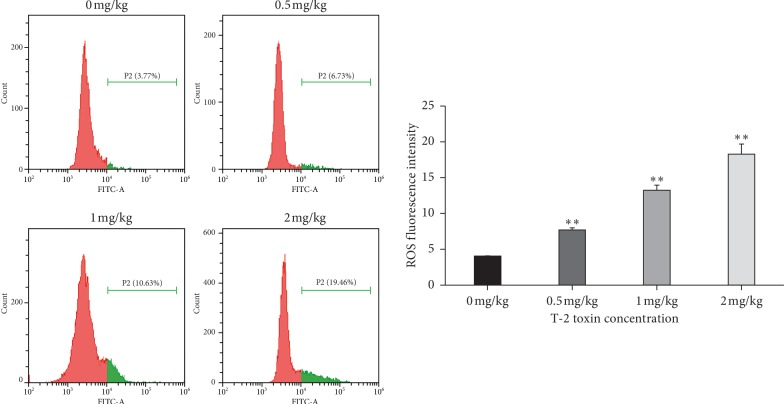
Effect of T-2 toxin exposure on concentrations of ROS in chickens' splenocytes. Intracellular ROS levels are determined using H2DCFDA staining. Data are expressed as mean ± sd (*n* = 6). ^*∗*^*P<*0.05, ^*∗∗*^*P<*0.01, as compared to control.

The effect of T-2 toxin exposure on concentrations of intracellular ROS in broiler chicken splenocytes was measured. Overall, DCFH-DA fluorescence and concentrations of intracellular ROS were significantly greater in splenocytes of broiler chickens exposed to T-2 toxin when compared to control exposed broiler chickens (Figure 4; *P<*0.01). Overall, these results suggest that the exposure of broiler chickens to T-2 toxin results in increased intracellular ROS in splenocytes.

### 3.5. Concentrations of Antioxidant Enzymes

**Figure 5:**
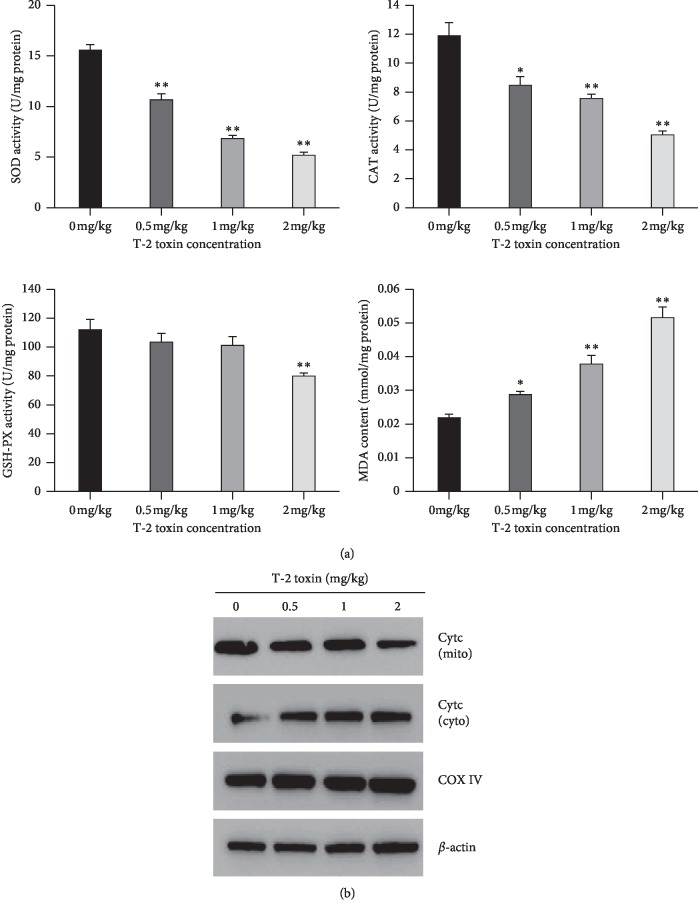
Effect of T-2 toxin exposure on antioxidative enzymes, MDA, and Cyt c in chickens' splenocytes. (a) Activity of SOD, CAT, GSH-PX, and MDA content in the spleen. (b) Mitochondrial and cytosolic cyt c in splenocytes of broiler chickens. Data are expressed as mean ± sd (*n* = 6). ^*∗*^*P<*0.05, ^*∗∗*^*P<*0.01, as compared to control.

Activities of SOD, CAT, and GSH-PX, and concentrations of MDA in splenocytes were measured by the use of commercial kits (Figure 5(a)). Activities of SOD and CAT significantly decreased in splenocytes of broiler chickens exposed to 1 and 2 mg/kg T-2 toxin when compared to control exposed chickens (*P<*0.01). In addition, the exposure of broiler chickens to 2 mg/kg T-2 toxin resulted in significant effects on GSH-PX activity when compared to control exposed chickens (*P<*0.01). Concentrations of MDA in splenocytes of broiler chickens exposed to T-2 toxin were significantly greater in all treatment groups when compared to control exposed chickens. Furthermore, it was observed by the use of Western blot that mitochondrial cyt c decreased in a dose-dependent manner whereas cytosolic cyt c increased (Figure 5(b)).

### 3.6. Concentrations of Splenic T-cell Subsets

To gain a greater understanding of the immune function of spleens of broiler chickens exposed to T-2 toxin, CD4+ and CD8+ T-cell counts were recorded (Table 2). Counts of CD4+ T cells decreased as the exposure of broiler chickens to T-2 toxin increased. In addition, a similar trend was observed for CD8+ T cells as counts decreased in the 0.5, 1, and 2 mg/kg groups when compared to control exposed broiler chickens. Furthermore, ratio of CD4+ to CD8+ T cells decreased as the exposure to T-2 toxin increased when compared to control exposed broiler chickens.

## 4. Discussion

It is well known that the spleen plays an important role in the body's immune response. In the current study, the condition of broiler chicken spleens was evaluated by the use of its relative weight and size. The relative weights of spleens collected from broiler chickens treated with T-2 toxin were less than those of control exposed broiler chickens, thus indicating that the atrophy of the spleen had occurred and could potentially lead to a weakened immune system. These results were consistent with previous work in which a diet supplemented with T-2 toxin reduced relative weight of spleens collected from yellow-feathered chickens [18]. Histopathological observation indicated that decrease in spleen relative weight might have been due to blockages by red pulp. Furthermore, histological changes might be due to the accumulation of B and T cells in splenic nodules and periarterial lymphatic sheath [19]. Similarly, we observed increased numbers of necrotic cells and spleen peripheral lymphocytes in groups of broiler chickens treated with T-2 toxin, suggesting plethoric necrosis of B and T lymphocytes which can lead to impaired immune functioning of the spleen.

Apoptosis, or programmed cell death, occurs in multicellular organisms, facilitates elimination of unnecessary cells, and thus functions to maintain tissue homeostasis. A number of studies have reported induction of apoptosis following the exposure to T-2 toxin. For example, Shinozuka et al. [20] observed differences in susceptibility of lymphocytes in lymphoid tissues to T-2 toxin, whereby lymphocytes of the thymus were most sensitive, especially in the cortex. Similarly, the exposure of turkeys to 3 mg/kg T-2 toxin resulted in increased apoptosis and histological changes in the spleen after 48 h exposure [21]. Conversely, no significant change in apoptotic cell counts in the spleen of three-week-old broiler chickens fed a diet of 1 mg/kg T-2 toxin was observed, whereas increased rates of apoptosis were observed in the thymus after 24 h exposure [22]. In the present study, we observed increased splenocyte apoptosis with increasing exposure to T-2 toxin, which was similar to previous findings [23]. These results suggest that splenocyte apoptosis can be induced by the exposure of broiler chickens to T-2 toxin.

Oxidative stress is characterized by increased concentrations of ROS, ultimately leading to increased oxidation and damage of DNA, lipids, and other macromolecules, eventually leading to apoptosis [24, 25]. As “redox messengers,” ROS is important in intracellular signaling and regulation, whereas excess ROS promotes cell death by activating apoptotic pathways [26]. In the present study, concentrations of ROS, cytosolic cyt c, and intracellular activities of SOD, CAT, and GSH-PX increased, whereas concentrations of MDA and mitochondrial cyt c decreased with increasing the exposure to T-2 toxin in the diet of broiler chickens. These results are consistent with previous work that found intracellular oxidative stress contributes to T-2 toxin associated toxicity. For example, the exposure of human cervical cancer cells to T-2 toxin resulted in DNA damage and increased rates of apoptosis induced by oxidative stress via the caspase pathway [6]. Furthermore, the exposure of fetal brain cells to T-2 toxin resulted in increased rates of apoptosis due to oxidative stress via the MAPK pathway [27]. Similarly, results of this study observed increased rates of oxidative stress and apoptosis in splenocytes following the exposure of broiler chickens to T-2 toxin.

The T cells CD4+ and CD8+ are important components of the acquired immune system. CD4+ cells, also known as helper T cells, influence the secretion of antibodies by B cells and regulate immune responses of other T cells, whereas CD8+ cells are major cytotoxic effector cells [28, 29]. Changes in the abundances or ratio of CD4+ to CD8+ T cells are reflective of the condition of the immune system whereby a high CD4+/CD8+ ratio indicates hyperactive cellular immune system and the inverse is related to immunosuppression [30]. Results of this study suggest that abundances of CD4+ T cells decreased as the exposure to T-2 toxin increased. In contrast, abundances of CD8+ T cells declined at low doses of T-2 toxin but increased at higher concentrations. The CD4+/CD8+ T-cell ratio decreased as T-2 toxin dose increased, indicating that splenic immune function might be impaired in broiler chickens. These results are similar to previous work in which exposure of broiler chickens to a mixture of toxins including 1 mg/kg T-2 toxin resulted in decreased abundances of CD4+ lymphocytes and CD4+/CD8+ T-cell ratio in peripheral blood lymphocytes [18]. Furthermore, Kamalavenkatesh et al. [31] demonstrated that chickens fed a diet containing 1 mg/kg T-2 toxin had fewer splenic CD4+ lymphocytes when compared to control exposed chickens [31]. In addition, Wilkins et al. [32] reported that radiation-induced apoptosis in total human lymphocyte subpopulations decreased as the CD4+/CD8+ ratio increased [32]. Overall, results indicate that T-2-toxin-induced splenocyte apoptosis leads to a reduction in the CD4+/CD8+ ratio and affects the immune system of broiler chickens negatively.

In summary, results of the current study suggest that the exposure of broiler chickens to T-2 toxin results in increased oxidative stress leading to increased splenocyte apoptosis and impairs immune function.

## Figures and Tables

**Table 1:** Nutrient and dietary analysis of feed fed to broiler chickens (%).

Ingredient		Nutrient levels	
Corn	60	Crude protein	20.83
Soybean meal (46%)	31.2	ME (MJ/kg)	12.15
Corn gluten meal	3	Calcium	0.97
Soybean oil	1.14	Available phosphorus	0.44
Calcium carbonate	0.95	Lysine	1.11
Calcium hydrophosphate	1.85	Methionine	0.48
L-Lysine hydrochloride	0.08	Methionine and cysteine	0.86
DL-Methionine	0.16	Threonine	0.78
Salt	0.5		
Choline chloride	0.15		
Vitamin and mineral premix	0.33		
Rice hull	0.64		
Total	100		

**Table 2:** Effects of T-2 toxin exposure on antigen expression of splenic T-cell subsets in broiler chickens. Data are expressed as mean ± sd (*n* = 6).

T-cell subsets	0 mg/kg	0.5 mg/kg	1 mg/kg	2 mg/kg
CD4+	10.76 ± 0.22^a^	10.21 ± 0.27^a^	9.76 ± 0.18^b^	8.29 ± 0.33^c^
CD8+	9.12 ± 0.12^a^	9.01 ± 0.23^a^	9.84 ± 0.28^b^	10.26 ± 0.17^c^
CD4+/CD8+	1.18 ± 0.09^a^	1.13 ± 0.17^a^	0.98 ± 0.08^b^	0.81 ± 0.11^c^

^a-c^Values with different superscripts are significantly different (*P<*0.05).

## Data Availability

The data used to support the findings of this study are included within the article.
